# Access to, and uptake of, antiretroviral therapy in a developing country with high HIV prevalence: a population-based cohort study in rural Uganda, 2004–2008

**DOI:** 10.1111/j.1365-3156.2012.02942.x

**Published:** 2012-07-30

**Authors:** Patrick Kazooba, Ivan Kasamba, Kathy Baisley, Billy N Mayanja, Dermot Maher

**Affiliations:** 1 MRC/UVRI Uganda Research Unit on AIDSEntebbe, Uganda; 2 London School of Hygiene and Tropical MedicineLondon, UK

**Keywords:** antiretroviral therapy, Uganda, access and uptake, cohort study

## Abstract

**Objectives:**

To investigate antiretroviral therapy (ART) uptake after its introduction in 2004 in a longitudinal population-based cohort and its nested clinical cohort in rural Uganda.

**Methods:**

A HIV serosurvey of all adults aged ≥15 years is conducted annually. Two intervals were selected for analysis. Interval 1 (November 2004–October 2006) provided 2 years of follow-up to prospectively evaluate access to HIV services. Interval 2 (November 2007–October 2008) was used to evaluate current coverage of services. Logistic regression was used to identify sociodemographic factors associated with ART screening within 2 years of diagnosis. ART coverage was assessed using Weibull survival models to estimate the numbers needing ART.

**Results:**

In Interval 1, 636 HIV-positive adults were resident and 295 (46.4%) knew their status. Of those, 248 (84.1%) were screened for ART within 2 years of diagnosis. After adjusting for age, those who were widowed, separated or never married were more likely to be screened than those who were married. In Interval 2, 575 HIV-positive adults were residents, 322 (56.0%) knew their status, 255 (44.3%) had been screened for ART and 189 (32.9%) had started ART. Estimated ART coverage was 66%.

**Conclusions:**

In this cohort, ART access and uptake is very high once people are diagnosed. Owing to intensive screening in the study clinic, nearly all participants who were eligible initiated ART. However, this is unlikely to reflect coverage in the general population, intensified efforts are needed to promote HIV testing, and ART screening and uptake are needed among those found to be HIV-positive.

## Introduction

Antiretroviral therapy (ART) has led to the improved health of many HIV-infected people and is a cost-effective intervention, as it makes HIV infection a chronic manageable disease, allowing HIV-infected people to live well and to be socially and economically active (Badri *et al.* 2006). While globally, the number of adults and children living with HIV increased from 29 million in 2001 to 33.4 million in 2008, the number of HIV-related deaths rose only from 1.9 million to 2 million over the same period, largely due to ART scale-up (UNAIDS/WHO, 2009).

There are many challenges in meeting the demand for ART in sub-Saharan Africa (Loewenson & McCoy 2004), the region most badly affected by HIV, with 70% of the world’s HIV-related deaths (UNAIDS/WHO, 2009). At the end of 2008, an estimated 44% of adults and children (nearly 3 million people) in need of ART in sub-Saharan Africa were receiving it (UNAIDS/WHO 2009). Barriers to ART access and uptake in Africa include misperceptions, distance to the ART providers and fear of side-effects of divorce or rejection by spouses, of stigma following HIV serostatus disclosure to friends and relatives, and of taking medicines for life (Murray *et al.* 2009).

Information on ART access and uptake is important in planning comprehensive HIV care services. Overcoming particular barriers to ART access among hard-to-reach populations, including those in rural areas (Zachariah *et al.* 2006), is critical if countries are to achieve universal access to ART. A longstanding population-based cohort in rural Uganda and its nested clinical cohort provided the opportunity to assess ART access and uptake and to investigate possible associated demographic factors.

## Methods

### Study setting

Adult HIV prevalence in Uganda fell from a peak of around 15% in 1991 to 5% in 2001 (Uganda STD/AIDS Control programme 2002) with recent signs of a possible resurgence (Uganda AIDS Commission 2007). An open general population cohort in rural southwest Uganda was established in 1989 for HIV surveillance, and now comprises approximately 20,000 residents of 25 neighbouring villages about 30 km from Lake Victoria. There are no tarmac roads, and access may be difficult during the rains. The population is ethnically homogeneous with most people belonging to the Baganda tribe and 15% being of Rwandese origin (Nakibinge *et al.* 2009). Half the population is under 15 years of age. HIV prevalence in the study area declined from 8.5% in 1990 to 6.2% in 1999/2000, but thereafter rose to 7.7% in 2004/2005 (Shafer *et al.* 2008).

### Population cohort and annual HIV serosurvey

Full details of the population cohort and annual HIV serosurvey have been published elsewhere (Mulder *et al.* 1994; Mbulaiteye *et al.* 2002). In brief, household surveys of consenting participants aged at least 13 years have been conducted annually since 1989, with all study village residents eligible for inclusion. Consenting residents are interviewed at home in the local language by trained survey staff and provide a blood sample for HIV testing. Survey rounds begin in November and end in October the following year. Participants are also encouraged to attend one of the five free voluntary counselling and testing (VCT) centres provided by the research programme in the study villages and at the study clinic. During the survey, participants are asked if they would like to receive their HIV test results, and if they have ever received their results in the past. Participants who indicate that they would like to receive their test result are referred to the VCT centres, where they are informed of their result and counselled. Diagnosis of HIV infection (i.e. having a test and learning the result) is the first step in access to ART.

### Clinical cohort

The clinical cohort has been described in detail elsewhere (Morgan *et al.* 1997). In brief, in 1990, a random selection of one-third of the HIV-positive adults identified in the first population cohort HIV serosurvey were invited to join a clinical cohort as prevalent cases. In addition, all adult HIV seroconverters identified during subsequent annual serosurveys were invited to enrol in the clinical cohort as HIV incident cases. HIV-negative controls randomly selected from the population cohort to match the age and gender of the HIV prevalent and incident cases, were also invited to enrol. Study clinicians were initially blinded to the participants’ HIV serostatus and were un-blinded in 2004 when ART was introduced. As participants who consent to the annual HIV serosurvey may opt not to know their HIV serostatus, some of those invited to enrol are unaware of their HIV serostatus and are therefore encouraged to access VCT at the centres in the study area and at the study clinic. Consenting individuals are enrolled in the clinical cohort and attend medical follow-up visits at the study clinic every 3 months. In January 2004, HIV care in the clinical cohort was extended to include ART provision for eligible and consenting participants.

Other HIV-positive individuals from the population cohort who present at the study clinic with symptoms and signs of HIV infection and those identified through the VCT centres are offered ART care, if eligible. These individuals are also invited to enrol in the clinical cohort. The study clinic is the only ART provider in the area (ART is not provided at the local government health centre or at the VCT centres). It is likely that very few individuals from the study population who are eligible for ART obtain it from other providers, as these are outside of the area (the nearest about 10 km away), and reaching them requires substantial efforts in time and transport costs.

### ART initiation

HIV-positive participants in the clinical cohort who know their HIV status undergo regular 3 monthly follow-up, including screening for ART eligibility according to national guidelines: CD4 count ≤200 cells/mm^3^ regardless of WHO clinical stage, WHO clinical stage 4 irrespective of CD4 count; persistent or recurrent WHO clinical stage 3 disease irrespective of CD4 count; and pregnant women with CD4 count ≤250 cells/mm^3^ (Uganda Ministry of Health 2003).

Intensive preparations for HIV-positive patients eligible for ART include three counselling visits, a medical examination and identification of a treatment adherence supporter. On starting ART, patients are seen at the clinic after 2 weeks, 4 weeks and then at 3-monthly intervals, or more often in case of interim illnesses or drug side-effects. First line treatment consists of a combination of zidovudine (AZT), lamivudine (3TC) and nevirapine (NVP), with the possibility of switching to stavudine (d4T) in case of AZT toxicity, and to efavirenz (EFZ) in case of NVP toxicity or concurrent tuberculosis treatment. Participants who are not yet eligible for ART start cotrimoxazole prophylaxis and receive an appointment for re-screening for ART eligibility.

### Laboratory methods

HIV serostatus was determined by two independent enzyme immunoassays (EIA) 5 (College of American Pathologists, 325 Waukegan Road, Northfield Illinois 60093-2750 USA) using a parallel set algorithm, with Western blot to resolve discordance between EIA results (Nunn *et al.* 1993). CD4 cell counts were measured using the FACS Count machine (Becton Dickinson, San Jose, CA, USA).

### Statistical methods

Data were analysed using Stata 10 (StataCorp, College Station, TX, USA). All adult participants (defined as aged ≥15 years) were included in the analysis if they had tested HIV-positive at any annual HIV survey, and were resident in the study area during the selected survey. Two intervals were selected for analysis: Interval 1 from November 2004 to October 2006 (i.e. the 2004/5 and 2005/6 surveys) and Interval 2 from November 2007 to October 2008 (i.e. the 2007/8 survey). As complete data on HIV services were not available after 2008, Interval 1 was chosen as a period after the early phase of ART introduction which allowed at least 2 years of follow-up to prospectively evaluate access to HIV care. Interval 2 was chosen as the most recent period for which complete information was available to estimate the current number who were HIV-infected, and who had accessed care and treatment.

Records were obtained from all VCT centres in the study villages of all participants who had received their HIV test results between January 2004 and the end of 2008. ART screening records from the clinical cohort were used to identify those participants who had received their HIV results from the study clinic, as all persons who receive their results in the clinic are screened for ART eligibility.

HIV diagnosis was defined as receiving one’s positive test result (either through a VCT centre or the study clinic). Individuals who reported receiving their results since testing HIV-positive in a serosurvey, but for whom there was no record at the VCT centre, were also considered to have been diagnosed. The date of diagnosis was defined as the earliest of the date of recorded VCT, ART screening or reported receipt of a positive result.

Interval 1 was used to explore factors associated with ART screening within 2 years of diagnosis. HIV-positive participants who had been diagnosed (i.e. received their positive test result) by end 2006 were followed prospectively to assess those who had been screened within 2 years of their diagnosis. Participants who died or moved away after their diagnosis were included in the analysis.

Cross-tabulations and chi square tests were conducted to assess associations between socio-demographic characteristics and being screened for ART within 2 years of diagnosis. Logistic regression was used to estimate crude and adjusted odd ratios (OR) and 95% confidence intervals (CI) for the associations between socio-demographic characteristics and ART screening. Age was included as an *a priori* confounder. Factors whose age-adjusted association with screening reached *P* < 0.20 were included in a multivariable model; those that remained associated at *P* < 0.10 were retained.

Interval 2 was used in calculating the proportion of HIV-infected participants still alive who had been diagnosed since the introduction of ART in 2004, and the proportion of those diagnosed who had been screened for ART eligibility. ART uptake was calculated as the proportion of those diagnosed and found eligible after screening who initiated ART. HIV-infected participants who had died or moved away before the 2007/8 HIV survey were excluded from the analysis.

Interval 2 was also used in estimating ART coverage relative to ART need, based on the 2003 Uganda Ministry of Health treatment guidelines (CD4 count threshold of 200 cells/mm^3^). Coverage is defined as the number of individuals receiving ART at a point in time divided by the number of individuals who are eligible to receive treatment at the same point in time (including those who are already receiving ART) (Mahy *et al.* 2010). A Weibull model was used to estimate age-specific mortality among all HIV-positive adults in the population cohort in the absence of ART. The Weibull regression parameters were used to construct an age-based model life table to calculate the conditional probability of survival to the age of ART need from the age at seroconversion, or from the age at the first positive HIV test in prevalent cases (Zaba *et al.* 2012). The date of seroconversion was calculated as the midpoint between the last documented negative HIV test and the first documented positive HIV test. Estimates of the number of participants needing ART at the end of 2008 were generated using survival time post-infection with the assumption that HIV-positive participants need ART approximately 3 years before otherwise expected death in the absence of ART (Schneider *et al.* 2004; Zwahlen 2008).

### Ethical approval

The study was part of a programme of research approved by the Science and Ethics Committee of the Uganda Virus Research Institute and the Uganda National Council of Science and Technology, and all participants gave informed consent.

## Results

During Interval 1 (the 2004/5 and 2005/6 surveys), 636 HIV-positive adults were resident in the study area, and by the end of 2006, 295 (46.4%) had been diagnosed (received their test results). Most (*N* = 201, 68.1%) had first received their positive result at the study clinic – the other 94 (31.9%) had received their result at a VCT centre (Table 1). However, 36 of those who reported receiving their results at a VCT centre had no confirmed record of diagnosis. The majority of those diagnosed were women (*N* = 178, 60.3%), aged between 25 and 44 years at the time of diagnosis, and had completed primary education or higher. Among those diagnosed, 248 (84.1%) had been screened for ART eligibility within 2 years of diagnosis. Of the 47 who had not been screened within 2 years, 10 (21.3%) had moved out of the study area after their diagnosis and 7 (14.9%) had died.

**Table 1:** Characteristics of 295 HIV-infected adults who were resident in the study area in Interval 1* and who knew their HIV status by end 2006

	*N* (%)
Gender	
Men	117 (39.7)
Women	178 (60.3)
Age (years) at diagnosis	
<25	30 (10.2
25–34	105 (35.6
35–44	106 (35.9)
45+	54 (18.3
Marital status at diagnosis	
Never Married	14 (4.7)
Married	136 (46.1)
Divorced/separated	77 (26.1)
Widowed	62 (21.0)
Not known	6 (2.0)
Education level at diagnosis	
None	34 (11.5)
Primary 1–4	61 (20.7)
Primary 5–7	114 (38.6)
Secondary or higher	54 (18.3)
Unknown	32 (10.8)
Tribe	
Baganda	191 (64.7)
Non-Baganda	95 (32.2)
Unknown	9 (3.1)
Location of first HIV diagnosis	
Study clinic	201 (68.1)
VCT centre	94 (31.9)
Has confirmed record of diagnosis	
Yes, from study clinic	240 (81.4)
Yes, from VCT centre	19 (6.4)
No (self report only)	36 (12.2)
Year of first diagnosis	
<2004	14 (4.7)
2004	188 (63.7)
2005	40 (13.6)
2006	53 (18.0)
Screened for ART eligibility within 2 years of diagnosis	
No	47 (15.9)
Yes	248 (84.1)
CD4 count category at first ART screening†	
<=250	143 (57.7)
251–600	67 (27.0)
>=601	38 (15.3)

ART, antiretroviral therapy; VCT, voluntary counselling and testing.

* Interval 1 is from November 2004 to October 2006 inclusive (the 2004/5 and 2005/6 HIV surveys).

† Among the 248 individuals screened.

Among the 295 HIV-positive participants in Interval 1 who were diagnosed by end 2006, there was some evidence in the unadjusted analysis that participants who were married were less likely to be screened for ART within 2 years of diagnosis (Table 2). No other sociodemographic factors were associated with screening in the unadjusted analysis. After adjusting for age as an a priori confounder, there was still some evidence of an association with marital status. Widowed, separated or never-married participants were more likely to be screened than married participants (adjusted OR = 2.72, 95% CI = 0.98–7.58, for widowed *vs.* married; adjusted OR = 4.97, 95% CI = 0.57–43.5 for never married *vs.* married). There was no evidence of an independent association between other the socio-demographic factors and screening for ART. Furthermore, the unadjusted and adjusted ORs were similar, indicating no important confounding of sociodemographic factors.

**Table 2:** Factors associated with being screened for ART initiation within 2 years, among HIV-infected adults who were resident in the study area in Interval 1* and knew their HIV status by end of 2006

	*n* screened/total HIV-positive & diagnosed (%)	Crude OR (95% CI)	Adjusted OR (95% CI)†
Gender		*P* = 0.66	*P* = 0.78
Men	97/117 (82.9)	1	1
Women	151/178 (84.8)	1.15 [0.61, 2.17]	1.10 [0.56, 2.17]
Age (years) at diagnosis		*P* = 0.70	*P* = 0.65
<25	23/30 (76.7)	1	1
25–34	88/105 (83.8)	1.58 [0.58, 4.25]	1.86 [0.65, 5.33]
35–44	91/106 (85.8)	1.85 [0.67, 5.05]	1.97 [0.66, 5.85]
45+	46/54 (85.2)	1.75 [0.56, 5.42]	2.06 [0.62, 6.88]
Marital status at diagnosis		*P* = 0.09	*P* = 0.08
Never married	13/14 (92.9)	3.37 [0.42, 26.9]	4.97 [0.57, 43.5]
Married	108/136 (79.4)	1	1
Divorced/separated	64/77 (83.1)	1.28 [0.62, 2.64]	1.23 [0.59, 2.56]
Widowed	57/62 (91.9)	2.96 [1.08, 8.07]	2.72 [0.98, 7.58]
Education at diagnosis		*P* = 0.49	*P* = 0.43
None	27/34 (79.4)	0.48 [0.15, 1.58]	0.44 [0.13, 1.50]
Primary 1–4	52/61 (85.3)	0.72 [0.24, 2.18]	0.69 [0.22, 2.11]
Primary 5–7	92/114 (80.7)	0.67 [0.20, 1.38]	0.49 [0.18, 1.32]
Secondary or higher	48/54 (88.9)	1	1
Tribe		*P* = 0.66	*P* = 0.79
Baganda	159/191 (83.2)	1	1
non-Baganda	81/95 (85.3)	1.16 [0.59, 2.30]	1.10 [0.54, 2.24]

ART, antiretroviral therapy; CI, confidence intervals; OR, odd ratios.

* Interval 1 is from November 2004 to October 2006 inclusive (the 2004/5 and 2005/6 HIV surveys).

† Adjusted for age and marital status.

**Figure 1:**
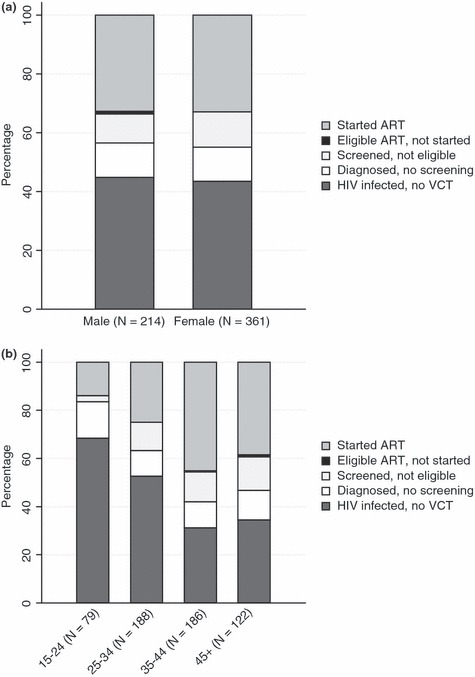
(a) HIV services coverage at end of 2008, among 575 HIV-infected adults who were alive and still resident in the study area in Interval 2^1^, by gender. (b) HIV services coverage at end of 2008, among 575 HIV-infected adults who were alive and still resident in the study area in Interval 2^1^, by age group. ^1^Interval 1 is from November 2007 to October 2008 inclusive (the 2007/8 HIV survey).

During Interval 2 (the 2007/8 survey), 575 adults had tested HIV-positive in any annual HIV survey and were still alive and resident in the study area. Of those, 322 (56.0%) had been diagnosed (received their test results) and knew that they were HIV-positive. By the end of 2008, 255 of those diagnosed (79.2%) had been screened for ART. Among those screened, 191 (74.9%) had ever been eligible to initiate ART, and of those who were eligible, 189 (99.0%) had initiated ART. The proportion of participants diagnosed, screened and started ART by gender and age group are shown in Figures 1a,b.

Among the 575 HIV-positive participants resident in Interval 2, it was estimated that 274 (48%) were in need of ART, of whom 189 (69%) had initiated treatment by end 2008 (Table 3). Coverage was slightly lower in males than females (64%*vs.* 72%, respectively). Coverage varied with age, from 55% in those aged 15–24 years to 86% in those aged 35–44 years. However, among those aged 45 years and above, coverage was 63%. Coverage also varied with marital status, with the highest coverage in widowed persons (91%) and the lowest among the currently married (58%).

**Table 3:** Estimated ART coverage at the end of 2008 among 575 HIV-infected adults who were alive and still resident in the study area in Interval 2*, by socio-demographic characteristics

	Number HIV-positive and resident	Number on ART	Estimated number needing ART	Estimated coverage (%)
Overall	575	189	274	69
Gender				
Men	214	70	109	64
Women	361	119	166	72
Age (years)				
15–24	79	11	20	55
25–34	188	47	81	58
35–44	186	84	98	86
45+	122	47	75	63
Current marital status				
Never Married	42	9	12	75
Married	288	80	138	58
Divorced /separated	158	57	77	74
Widowed	87	43	47	91
Current education				
None	38	14	20	70
Primary 1–4	161	38	49	78
Primary 5–7	245	73	115	63
Secondary or higher	131	42	62	68
Tribe				
Baganda	404	131	191	69
non-Baganda	171	58	83	70

ART, antiretroviral therapy.

* Interval 2 is from November 2007 to October 2008 inclusive (the 2007/8 HIV survey).

## Discussion

Based on 2003 Uganda national treatment guidelines (Uganda Ministry of Health 2003), estimated overall ART coverage in our rural programme was 69%, reflecting a high level of ART access and uptake, once people were diagnosed with HIV infection, but a low level of HIV diagnosis. Diagnosis of HIV infection was the main bottleneck in the successive steps that determine overall ART coverage: diagnosis of HIV infection, screening for ART eligibility and receiving ART. Our cross-sectional analysis of HIV-positive participants who were still resident in 2008 shows that slightly over half (56%) had been diagnosed (i.e. knew their HIV status). However, among those who were diagnosed, most (79%) had been screened for ART eligibility. Our prospective analysis of HIV-positive participants who were resident in 2004–2006 showed a similar picture – less than half had been diagnosed by end 2006, but among those who were diagnosed, 84% had been screened within 2 years of diagnosis.

The estimated 69% ART coverage in our programme is higher than the estimated 53% coverage for Uganda nationally in 2009 (WHO/UNAIDS/UNICEF, 2010) using the previous WHO guidelines that recommended ART initiation at CD4 count ≤200 cells/mm^3^ (WHO 2006). The results from our research programme with a long presence in the local community and an intensive screening approach represent ‘best practice’, and are therefore unlikely to be representative of those obtained nationally through routine service provision. A comparative analysis of ART access in four population-based HIV cohort studies in Africa found that coverage was higher in Masaka than in sites in Malawi, Tanzania and Zimbabwe (Wringe *et al.* 2012). A study in Malawi showed that those accessing care are those with the most advanced disease and the highest mortality in absence of treatment (Jahn *et al.* 2008). In our cohort, most people who were diagnosed with HIV attended the study clinic, and so were screened for ART eligibility at the time of diagnosis. However, in the general population, both HIV diagnosis and access to ART screening may be important bottlenecks in the coverage of HIV services. Further pressure on ART coverage will come with changes in policy, recommending that people living with HIV should start on ART with a CD4 count of 350 or 500 cells/mm^3^. Using a CD4 count of 350 as the threshold for treatment, an estimated 73% of HIV-positive individuals in our cohort in 2008 would be in need of ART, and coverage would be only 45%.

In designing interventions to improve ART coverage, it is important to know about the demographic factors affecting ART access and uptake, but there have been few studies. We have identified various factors associated with uptake of ART screening after diagnosis that could be targeted in developing strategies to improve HIV care programmes. After adjusting for age, we found that among people who had learned their HIV-positive status, married people were less likely to be screened for ART within 2 years than those who were never married, or who had been separated, divorced or widowed. Studies have demonstrated spousal barriers in the uptake of HIV services (De Paoli *et al.* 2004; Muula & Kataika 2008). Neither disclosure nor domestic violence was examined, but these concerns could contribute to the lower uptake of ART and screening services among married couples. The relatively low uptake of ART services among young people and married people indicates the scope for efforts to provide services that meet the needs of these groups. The need for ART is high among people with HIV who are married, in whom HIV prevalence is high (Government of Uganda 2010).

The main strength of our study is the ability to link population-level HIV data with information from the only ART provider in the study area, enabling us to assess the proportion of HIV-infected persons who have been diagnosed, screened and started on ART. Information on age-specific mortality and HIV incidence also enables us to estimate ART need among the HIV-infected population, providing a denominator, against which ART coverage can be calculated, disaggregated by socio-demographic variables of interest.

A limitation is that, unlike data collection in the clinical cohort study, data in the VCT centres are not routinely collected for analysis purposes, and so, the VCT data may be incomplete. Hence, we may have underestimated the proportion of participants who know their HIV status. An additional limitation is that our estimate of current access to HIV services is based on HIV-positive adults who were still alive during 2007/8, the most recent survey for which complete data on services was available. Thus, we will have missed people who died or moved away before the 2007/8 survey, and may be overestimating the proportion of adults in the study area who access HIV services.

The roll-out of free ART in Uganda started in 2004 and achieved an estimated 39% national coverage by 2009 (WHO/UNAIDS/UNICEF 2010) based on 2010 WHO guidelines that recommend ART initiation at a CD4 count of 350 cells/mm^3^ (World Health Organization 2010). The standard definition of ART coverage depends on set criteria for unmet need (Mahy *et al.* 2010), enabling comparison of coverage estimates that use the same eligibility criteria. However, the change in the WHO recommended CD4 count threshold for ART initiation from 200 to 350 cells/mm^3^ results in changes in coverage estimates, which may decrease even as countries enrol greater numbers of patients onto ART. Measures to complement the existing UNGASS definition of ART coverage may be useful, e.g. the enrolment ratio, in which the numerator is the number of individuals starting ART in a given year, and the denominator is the number of individuals becoming eligible for ART in the same year, according to the ART guidelines that are in place during that year (Johnson & Boulle 2011). This ratio of ART initiation to HIV progression provides a better reflection of recent programme performance and a robust measure that is less sensitive to changes in ART eligibility criteria.

## Conclusions

Overall, ART coverage after 4 years of this rural programme was 69%, reflecting high ART access and uptake once people are diagnosed with HIV infection, but a low level of HIV diagnosis. At least half of HIV-infected participants may not know their status, even though in this setting, people have more opportunities to learn their status than in other rural areas in Uganda. However, once diagnosed, nearly all participants who were eligible for ART were started on treatment, owing to the intensive screening procedures in the study clinic. This very high uptake of ART is unlikely to reflect coverage in the general population. Intensified efforts are needed to promote HIV testing and ART screening and uptake among those found to be HIV-positive.
